# The Physiology of the Visual Purple

**Published:** 1881-05

**Authors:** 


### THE PHYSIOLOGY OF THE VISUAL PURPLE.

In the May number of the *New York Medical Journal*, Dr. William C. Ayres concludes a series of articles on the visual purple. We know, he says, that the purple is a photo-chemical substance which is sensitive to light, and that its seat is in the outer segments of the rods, whereas it is never found in the cones. The cones, on the other hand, being the only elements found in the fovea centralis, we are forced to the conclusion that distinct vision, both for objects and for colors, is independent of its existence. In the higher classes of animals it is sensitive to light, but in some deep-sea fishes, cephalopods, etc., it has its seat in the rods, but is no longer sensitive to light, although it has the same color as before. In another variety of fishes, the black fish, for instance, it is purple in color, but resides in the slender offshoots of the pigment cells which run in between the rods and cones. Where it is not sensitive to light, the optical structure of the eye is very defective, and any benefit of a sensitive compound would not be appreciated, and it therefore does its work as a stable pigment. It is an *albuminoid compound* and is a *secretion of the pigment epithelial cells of the retina*, but *this secretion is not controlled by any one of the larger nerve trunks* which have a part to play in the functions of the eye. We know of no drug which can diminish its secretion, but *pilocarpine and muscarine greatly increase it.* We know that a solution of the purple does not destroy the most chemical rays, as those of the violet and ultra-violet light, but, on the contrary, the yellow, which is not so deep in color as the purple, cuts off these waves completely. We know also that when the purple is in excess in the retina there must be the greatest chemical effect possible, and that if the light come into such an eye it can not see or is overpowered. Thus, when the eye has been for a long time in the dark and comes into the light, it is dazzled. Again, when the yellow is in excess, there is a minimum of chemical effect capable of being produced in the retina, and the eye does not see either, as when we pass from a bright light into a darker room. *The purple is a conservation compound which is placed in the eye as a matter of protection*, and which enables it to perform its duty under the most varied circumstances. In eyes which are not subjected to such variations, it is no longer sensitive to light, but it preserves all of its characteristics. The idea of using it for medico-legal purposes is impracticable.

